# 4,4′-Dimeth­oxy-2,2′-[2,2-dimethyl­propane-1,3-diylbis(nitrilo­methanylyl­idene)]diphenol

**DOI:** 10.1107/S1600536811004776

**Published:** 2011-02-12

**Authors:** Hadi Kargar, Reza Kia, Elham Pahlavani, Muhammad Nawaz Tahir

**Affiliations:** a Chemistry Department, Payame Noor University, Tehran 19395-4697, I. R. of Iran; b X-ray Crystallography Laboratory, Plasma Physics Research Center, Science and Research Branch, Islamic Azad University, Tehran, Iran; c Department of Physics, University of Sargodha, Punjab, Pakistan

## Abstract

In the title compound, C_21_H_26_N_2_O_4_, the dihedral angle between the substituted benzene rings is 30.47 (15) °. Two strong intra­molecular O—H⋯N hydrogen bonds generate two *S*(6) ring motifs.

## Related literature

For hydrogen-bond motifs, see: Bernstein *et al.* (1995 ▶). For related structures, see: Kargar *et al.* (2009 ▶, 2010 ▶).
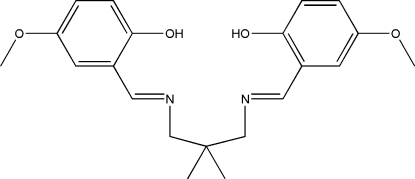


## Experimental

### 

#### Crystal data

C_21_H_26_N_2_O_4_
*M* *_r_* = 370.44
Monoclinic, 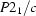

*a* = 10.660 (2) Å
*b* = 21.742 (4) Å
*c* = 9.2767 (19) Å
β = 108.03 (3)°
*V* = 2044.5 (7) Å^3^
*Z* = 4
Mo *K*α radiation
μ = 0.08 mm^−1^
*T* = 296 K
0.23 × 0.15 × 0.08 mm

#### Data collection

Stoe IPDS 2T Image Plate diffractometer
Absorption correction: multi-scan (*MULABS* in *PLATON*; Blessing, 1995 ▶) *T*_min_ = 0.965, *T*_max_ = 1.000
7094 measured reflections
3375 independent reflections
967 reflections with *I* > 2σ(*I*)
*R*_int_ = 0.054

#### Refinement

*R*[*F* ^2^ > 2σ(*F* ^2^)] = 0.031
*wR*(*F* ^2^) = 0.058
*S* = 0.57
3375 reflections
246 parameters
H-atom parameters constrained
Δρ_max_ = 0.08 e Å^−3^
Δρ_min_ = −0.11 e Å^−3^

### 

Data collection: *X-AREA* (Stoe & Cie, 2009 ▶); cell refinement: *X-AREA*; data reduction: *X-AREA*; program(s) used to solve structure: *SHELXTL* (Sheldrick, 2008 ▶); program(s) used to refine structure: *SHELXTL*; molecular graphics: *SHELXTL*; software used to prepare material for publication: *SHELXTL* and *PLATON* (Spek, 2009 ▶).

## Supplementary Material

Crystal structure: contains datablocks global, I. DOI: 10.1107/S1600536811004776/tk2716sup1.cif

Structure factors: contains datablocks I. DOI: 10.1107/S1600536811004776/tk2716Isup2.hkl

Additional supplementary materials:  crystallographic information; 3D view; checkCIF report

## Figures and Tables

**Table 1:** Hydrogen-bond geometry (Å, °)

*D*—H⋯*A*	*D*—H	H⋯*A*	*D*⋯*A*	*D*—H⋯*A*
O1—H1⋯N1	0.81	1.88	2.593 (3)	147
O2—H2⋯N2	0.83	1.90	2.604 (3)	143

**Fig. 1.**
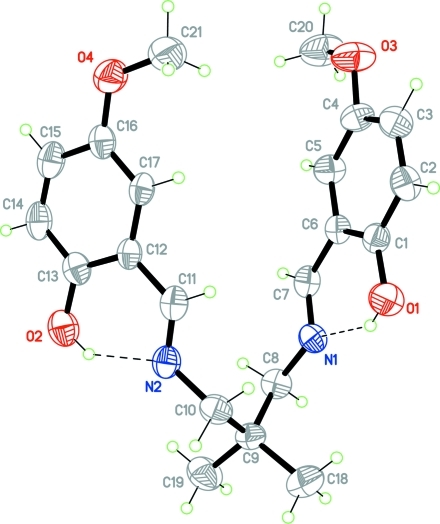
The molecular structure of the title compound, showing 30% probability displacement ellipsoids and the atomic numbering. Intramolecular hydrogen bonds are drawn as dashed lines.

## supplementary crystallographic information

### Comment

Schiff base ligands are one of the most prevalent systems in coordination chemistry. As part of a general study of potentially tetradenate Schiff bases (Kargar *et al.*, 2009; Kargar *et al.* 2010), we have determined the crystal structure of the title compound.

The asymmetric unit of the title compound, Fig. 1, comprises a potentially tetradenate Schiff base ligand. The bond lengths are comparable to previously reported structures (Kargar *et al.*, 2009, Kargar *et al.*, 2010). The dihedral angle between the two benzene rings is 30.47 (15) °. Strong intramolecular O—H···N hydrogen bonds (Table 1) generate two *S(6)* ring motifs (Bernstein *et al.*, 1995).

### Experimental

The title compound was synthesized by adding 5-methoxy-salicylaldehyde (4 mmol) to a solution of 2,2-dimethyl-1,3-propanediamine (2 mmol) in ethanol (20 ml). The mixture was refluxed with stirring for 30 min. The resultant yellow solution was filtered. Yellow crystals were obtained by slow evaporation of its ethanol solution at room temperature over several days.

### Refinement

H atoms of the hydroxy groups were located in a difference Fourier map and constrained at those positions with *U*_iso_(H) = 1.5 *U*_eq_(O), see Table 1 for distances. The remaining H atoms were positioned geometrically with C—H = 0.93–0.97 Å and included in a riding model approximation with *U*_iso_ (H) = 1.2 or 1.5 *U*_eq_ (C). A rotating group model was used only for the methyl groups of the methoxy substituents.

### Crystal data

**Table:**

C_21_H_26_N_2_O_4_	*F*(000) = 792
*M**_r_* = 370.44	*D*_x_ = 1.204 Mg m^−^^3^
Monoclinic, *P*2_1_/*c*	Mo *K*α radiation, λ = 0.71073 Å
Hall symbol: -P 2ybc	Cell parameters from 3220 reflections
*a* = 10.660 (2) Å	θ = 2.0–24.2°
*b* = 21.742 (4) Å	µ = 0.08 mm^−^^1^
*c* = 9.2767 (19) Å	*T* = 296 K
β = 108.03 (3)°	Plate, yellow
*V* = 2044.5 (7) Å^3^	0.23 × 0.15 × 0.08 mm
*Z* = 4	

### Data collection

**Table:**

Stoe IPDS 2T Image Plate diffractometer	3375 independent reflections
Radiation source: fine-focus sealed tube	967 reflections with *I* > 2σ(*I*)
graphite	*R*_int_ = 0.054
Detector resolution: 0.15 mm pixels mm^-1^	θ_max_ = 25.0°, θ_min_ = 2.0°
ω scans	*h* = −12→12
Absorption correction: multi-scan (*MULABS* in *PLATON*; Blessing, 1995)	*k* = −22→25
*T*_min_ = 0.965, *T*_max_ = 1.000	*l* = −11→10
7094 measured reflections	

### Refinement

**Table:**

Refinement on *F*^2^	Primary atom site location: structure-invariant direct methods
Least-squares matrix: full	Secondary atom site location: difference Fourier map
*R*[*F*^2^ > 2σ(*F*^2^)] = 0.031	Hydrogen site location: inferred from neighbouring sites
*wR*(*F*^2^) = 0.058	H-atom parameters constrained
*S* = 0.57	*w* = 1/[σ^2^(*F*_o_^2^) + (0.0172*P*)^2^] where *P* = (*F*_o_^2^ + 2*F*_c_^2^)/3
3375 reflections	(Δ/σ)_max_ = 0.001
246 parameters	Δρ_max_ = 0.08 e Å^−^^3^
0 restraints	Δρ_min_ = −0.11 e Å^−^^3^

### Special details

**Table:**

Geometry. All e.s.d.'s (except the e.s.d. in the dihedral angle between two l.s. planes) are estimated using the full covariance matrix. The cell e.s.d.'s are taken into account individually in the estimation of e.s.d.'s in distances, angles and torsion angles; correlations between e.s.d.'s in cell parameters are only used when they are defined by crystal symmetry. An approximate (isotropic) treatment of cell e.s.d.'s is used for estimating e.s.d.'s involving l.s. planes.
Refinement. Refinement of *F*^2^ against ALL reflections. The weighted *R*-factor *wR* and goodness of fit *S* are based on *F*^2^, conventional *R*-factors *R* are based on *F*, with *F* set to zero for negative *F*^2^. The threshold expression of *F*^2^ > σ(*F*^2^) is used only for calculating *R*-factors(gt) *etc*. and is not relevant to the choice of reflections for refinement. *R*-factors based on *F*^2^ are statistically about twice as large as those based on *F*, and *R*- factors based on ALL data will be even larger.

### Fractional atomic coordinates and isotropic or equivalent isotropic displacement parameters (Å^2^)

**Table:**

	*x*	*y*	*z*	*U*_iso_*/*U*_eq_	
O1	−0.05846 (17)	0.01529 (7)	0.75824 (19)	0.0807 (6)	
H1	−0.0997	0.0250	0.6727	0.121*	
O2	−0.1706 (2)	0.31943 (8)	0.4769 (2)	0.0874 (6)	
H2	−0.2189	0.2892	0.4703	0.131*	
O3	0.4634 (2)	0.07957 (10)	0.8988 (2)	0.1064 (8)	
O4	0.3081 (2)	0.30533 (10)	0.9255 (3)	0.0936 (7)	
N1	−0.1073 (3)	0.07562 (9)	0.5057 (3)	0.0629 (7)	
N2	−0.2263 (3)	0.21006 (10)	0.5539 (3)	0.0662 (7)	
C1	0.0688 (3)	0.03241 (12)	0.7858 (4)	0.0601 (8)	
C2	0.1605 (3)	0.01492 (12)	0.9224 (3)	0.0685 (8)	
H2A	0.1336	−0.0083	0.9917	0.082*	
C3	0.2893 (3)	0.03161 (12)	0.9549 (3)	0.0750 (9)	
H3A	0.3502	0.0195	1.0460	0.090*	
C4	0.3300 (3)	0.06678 (14)	0.8524 (4)	0.0720 (9)	
C5	0.2413 (3)	0.08523 (12)	0.7179 (3)	0.0705 (9)	
H5A	0.2691	0.1088	0.6498	0.085*	
C6	0.1091 (3)	0.06829 (11)	0.6844 (3)	0.0557 (8)	
C7	0.0141 (3)	0.08981 (11)	0.5447 (3)	0.0621 (8)	
H7A	0.0435	0.1151	0.4811	0.075*	
C8	−0.1960 (3)	0.10405 (11)	0.3705 (3)	0.0689 (8)	
H8A	−0.2106	0.0760	0.2856	0.083*	
H8B	−0.1554	0.1410	0.3468	0.083*	
C9	−0.3286 (3)	0.12057 (12)	0.3915 (3)	0.0647 (8)	
C10	−0.3096 (2)	0.15556 (12)	0.5410 (3)	0.0686 (8)	
H10A	−0.3951	0.1680	0.5473	0.082*	
H10B	−0.2698	0.1282	0.6255	0.082*	
C11	−0.1179 (3)	0.21062 (13)	0.6614 (3)	0.0647 (9)	
H11A	−0.0969	0.1777	0.7287	0.078*	
C12	−0.0261 (3)	0.26170 (13)	0.6810 (3)	0.0550 (8)	
C13	−0.0544 (3)	0.31384 (15)	0.5877 (3)	0.0673 (9)	
C14	0.0387 (4)	0.36057 (13)	0.6113 (4)	0.0794 (11)	
H14A	0.0205	0.3954	0.5500	0.095*	
C15	0.1556 (4)	0.35591 (14)	0.7224 (4)	0.0804 (10)	
H15A	0.2167	0.3876	0.7359	0.096*	
C16	0.1861 (4)	0.30500 (14)	0.8166 (4)	0.0677 (9)	
C17	0.0946 (3)	0.25820 (12)	0.7948 (3)	0.0628 (8)	
H17A	0.1140	0.2238	0.8573	0.075*	
C18	−0.4084 (2)	0.06177 (12)	0.3967 (3)	0.0982 (10)	
H18A	−0.3598	0.0366	0.4803	0.147*	
H18B	−0.4234	0.0393	0.3038	0.147*	
H18C	−0.4916	0.0729	0.4092	0.147*	
C19	−0.4063 (3)	0.16042 (12)	0.2556 (3)	0.0975 (10)	
H19A	−0.3572	0.1971	0.2522	0.146*	
H19B	−0.4901	0.1713	0.2666	0.146*	
H19C	−0.4199	0.1376	0.1634	0.146*	
C20	0.5161 (3)	0.11165 (14)	0.7998 (4)	0.1184 (13)	
H20A	0.6098	0.1155	0.8448	0.178*	
H20B	0.4771	0.1518	0.7810	0.178*	
H20C	0.4974	0.0896	0.7059	0.178*	
C21	0.3454 (3)	0.25225 (13)	1.0160 (3)	0.1204 (13)	
H21A	0.4318	0.2581	1.0868	0.181*	
H21B	0.3463	0.2175	0.9525	0.181*	
H21C	0.2834	0.2450	1.0703	0.181*	

### Atomic displacement parameters (Å^2^)

**Table:**

	*U*^11^	*U*^22^	*U*^33^	*U*^12^	*U*^13^	*U*^23^
O1	0.0710 (15)	0.0916 (13)	0.0825 (14)	−0.0025 (12)	0.0279 (14)	0.0249 (11)
O2	0.1028 (17)	0.0767 (14)	0.0822 (16)	0.0121 (13)	0.0279 (14)	0.0188 (12)
O3	0.0738 (17)	0.154 (2)	0.0852 (18)	−0.0283 (16)	0.0154 (16)	0.0102 (14)
O4	0.0945 (18)	0.0784 (16)	0.0994 (19)	−0.0159 (14)	0.0174 (16)	0.0104 (14)
N1	0.0633 (17)	0.0646 (15)	0.0623 (18)	0.0079 (15)	0.0216 (16)	0.0016 (13)
N2	0.078 (2)	0.0597 (16)	0.0666 (19)	0.0014 (16)	0.0305 (16)	−0.0021 (14)
C1	0.060 (2)	0.0550 (19)	0.070 (2)	0.0004 (17)	0.027 (2)	−0.0008 (17)
C2	0.081 (2)	0.070 (2)	0.062 (2)	0.006 (2)	0.034 (2)	0.0144 (17)
C3	0.076 (3)	0.085 (2)	0.063 (2)	0.003 (2)	0.021 (2)	0.0031 (18)
C4	0.061 (2)	0.084 (2)	0.073 (3)	−0.015 (2)	0.024 (2)	−0.007 (2)
C5	0.069 (2)	0.082 (2)	0.064 (2)	−0.0074 (19)	0.025 (2)	0.0053 (18)
C6	0.062 (2)	0.0521 (18)	0.059 (2)	0.0031 (17)	0.027 (2)	0.0024 (16)
C7	0.079 (2)	0.0551 (19)	0.063 (2)	−0.0008 (19)	0.036 (2)	0.0015 (16)
C8	0.072 (2)	0.077 (2)	0.060 (2)	0.0013 (18)	0.025 (2)	−0.0030 (17)
C9	0.058 (2)	0.0713 (19)	0.061 (2)	−0.0044 (18)	0.0138 (19)	−0.0057 (17)
C10	0.058 (2)	0.082 (2)	0.071 (2)	0.0058 (18)	0.0274 (18)	−0.0003 (17)
C11	0.086 (3)	0.058 (2)	0.062 (2)	0.003 (2)	0.039 (2)	−0.0006 (17)
C12	0.071 (2)	0.0477 (18)	0.053 (2)	0.0033 (18)	0.0293 (19)	−0.0010 (17)
C13	0.080 (3)	0.067 (2)	0.061 (2)	0.012 (2)	0.031 (2)	0.001 (2)
C14	0.112 (3)	0.051 (2)	0.089 (3)	0.004 (2)	0.050 (3)	0.017 (2)
C15	0.105 (3)	0.057 (2)	0.092 (3)	−0.007 (2)	0.048 (3)	0.002 (2)
C16	0.086 (3)	0.057 (2)	0.066 (2)	0.007 (2)	0.032 (2)	0.0060 (19)
C17	0.082 (2)	0.0454 (19)	0.065 (2)	−0.0025 (19)	0.029 (2)	0.0042 (16)
C18	0.085 (2)	0.099 (2)	0.102 (3)	−0.023 (2)	0.017 (2)	−0.015 (2)
C19	0.093 (2)	0.108 (2)	0.078 (2)	0.017 (2)	0.006 (2)	0.012 (2)
C20	0.082 (3)	0.161 (3)	0.113 (3)	−0.043 (2)	0.032 (2)	0.014 (2)
C21	0.114 (3)	0.098 (3)	0.119 (3)	−0.014 (2)	−0.008 (2)	0.028 (2)

### Geometric parameters (Å, °)

**Table:**

O1—C1	1.353 (3)	C9—C19	1.543 (3)
O1—H1	0.8074	C9—C18	1.544 (3)
O2—C13	1.348 (3)	C10—H10A	0.9700
O2—H2	0.8251	C10—H10B	0.9700
O3—C4	1.381 (3)	C11—C12	1.454 (3)
O3—C20	1.402 (3)	C11—H11A	0.9300
O4—C16	1.377 (3)	C12—C17	1.391 (3)
O4—C21	1.410 (3)	C12—C13	1.401 (3)
N1—C7	1.269 (3)	C13—C14	1.390 (3)
N1—C8	1.455 (3)	C14—C15	1.353 (4)
N2—C11	1.272 (3)	C14—H14A	0.9300
N2—C10	1.463 (3)	C15—C16	1.385 (3)
C1—C6	1.389 (3)	C15—H15A	0.9300
C1—C2	1.392 (3)	C16—C17	1.380 (3)
C2—C3	1.360 (3)	C17—H17A	0.9300
C2—H2A	0.9300	C18—H18A	0.9600
C3—C4	1.390 (3)	C18—H18B	0.9600
C3—H3A	0.9300	C18—H18C	0.9600
C4—C5	1.371 (3)	C19—H19A	0.9600
C5—C6	1.395 (3)	C19—H19B	0.9600
C5—H5A	0.9300	C19—H19C	0.9600
C6—C7	1.453 (3)	C20—H20A	0.9600
C7—H7A	0.9300	C20—H20B	0.9600
C8—C9	1.528 (3)	C20—H20C	0.9600
C8—H8A	0.9700	C21—H21A	0.9600
C8—H8B	0.9700	C21—H21B	0.9600
C9—C10	1.539 (3)	C21—H21C	0.9600
			
C1—O1—H1	108.8	N2—C11—C12	121.2 (3)
C13—O2—H2	112.9	N2—C11—H11A	119.4
C4—O3—C20	118.6 (3)	C12—C11—H11A	119.4
C16—O4—C21	117.4 (2)	C17—C12—C13	119.0 (3)
C7—N1—C8	118.4 (2)	C17—C12—C11	118.8 (3)
C11—N2—C10	116.9 (3)	C13—C12—C11	122.2 (3)
O1—C1—C6	121.9 (3)	O2—C13—C14	119.8 (3)
O1—C1—C2	118.5 (3)	O2—C13—C12	121.0 (3)
C6—C1—C2	119.5 (3)	C14—C13—C12	119.2 (3)
C3—C2—C1	120.3 (3)	C15—C14—C13	120.6 (3)
C3—C2—H2A	119.8	C15—C14—H14A	119.7
C1—C2—H2A	119.8	C13—C14—H14A	119.7
C2—C3—C4	120.1 (3)	C14—C15—C16	121.5 (3)
C2—C3—H3A	119.9	C14—C15—H15A	119.3
C4—C3—H3A	119.9	C16—C15—H15A	119.3
C5—C4—O3	125.3 (3)	O4—C16—C17	125.1 (3)
C5—C4—C3	120.7 (3)	O4—C16—C15	116.3 (3)
O3—C4—C3	114.0 (3)	C17—C16—C15	118.6 (3)
C4—C5—C6	119.3 (3)	C16—C17—C12	121.1 (3)
C4—C5—H5A	120.3	C16—C17—H17A	119.4
C6—C5—H5A	120.3	C12—C17—H17A	119.4
C1—C6—C5	120.0 (3)	C9—C18—H18A	109.5
C1—C6—C7	120.5 (3)	C9—C18—H18B	109.5
C5—C6—C7	119.4 (3)	H18A—C18—H18B	109.5
N1—C7—C6	123.0 (3)	C9—C18—H18C	109.5
N1—C7—H7A	118.5	H18A—C18—H18C	109.5
C6—C7—H7A	118.5	H18B—C18—H18C	109.5
N1—C8—C9	111.7 (2)	C9—C19—H19A	109.5
N1—C8—H8A	109.3	C9—C19—H19B	109.5
C9—C8—H8A	109.3	H19A—C19—H19B	109.5
N1—C8—H8B	109.3	C9—C19—H19C	109.5
C9—C8—H8B	109.3	H19A—C19—H19C	109.5
H8A—C8—H8B	107.9	H19B—C19—H19C	109.5
C8—C9—C10	111.2 (2)	O3—C20—H20A	109.5
C8—C9—C19	108.1 (2)	O3—C20—H20B	109.5
C10—C9—C19	110.3 (2)	H20A—C20—H20B	109.5
C8—C9—C18	110.4 (2)	O3—C20—H20C	109.5
C10—C9—C18	107.6 (2)	H20A—C20—H20C	109.5
C19—C9—C18	109.2 (2)	H20B—C20—H20C	109.5
N2—C10—C9	112.4 (2)	O4—C21—H21A	109.5
N2—C10—H10A	109.1	O4—C21—H21B	109.5
C9—C10—H10A	109.1	H21A—C21—H21B	109.5
N2—C10—H10B	109.1	O4—C21—H21C	109.5
C9—C10—H10B	109.1	H21A—C21—H21C	109.5
H10A—C10—H10B	107.9	H21B—C21—H21C	109.5
			
O1—C1—C2—C3	−179.5 (2)	C11—N2—C10—C9	−116.9 (3)
C6—C1—C2—C3	−1.5 (4)	C8—C9—C10—N2	54.6 (3)
C1—C2—C3—C4	0.6 (4)	C19—C9—C10—N2	−65.3 (3)
C20—O3—C4—C5	−2.9 (4)	C18—C9—C10—N2	175.7 (2)
C20—O3—C4—C3	176.3 (3)	C10—N2—C11—C12	177.96 (19)
C2—C3—C4—C5	0.2 (4)	N2—C11—C12—C17	−176.1 (3)
C2—C3—C4—O3	−179.0 (3)	N2—C11—C12—C13	3.0 (4)
O3—C4—C5—C6	179.1 (3)	C17—C12—C13—O2	−179.4 (2)
C3—C4—C5—C6	−0.1 (4)	C11—C12—C13—O2	1.5 (4)
O1—C1—C6—C5	179.6 (2)	C17—C12—C13—C14	0.1 (3)
C2—C1—C6—C5	1.7 (4)	C11—C12—C13—C14	−179.0 (2)
O1—C1—C6—C7	1.1 (4)	O2—C13—C14—C15	179.7 (3)
C2—C1—C6—C7	−176.8 (2)	C12—C13—C14—C15	0.2 (4)
C4—C5—C6—C1	−0.9 (4)	C13—C14—C15—C16	−0.4 (5)
C4—C5—C6—C7	177.6 (3)	C21—O4—C16—C17	3.8 (4)
C8—N1—C7—C6	174.6 (2)	C21—O4—C16—C15	−175.7 (2)
C1—C6—C7—N1	−2.5 (4)	C14—C15—C16—O4	179.9 (3)
C5—C6—C7—N1	179.0 (3)	C14—C15—C16—C17	0.3 (4)
C7—N1—C8—C9	−140.7 (2)	O4—C16—C17—C12	−179.6 (2)
N1—C8—C9—C10	48.8 (3)	C15—C16—C17—C12	−0.1 (4)
N1—C8—C9—C19	170.1 (2)	C13—C12—C17—C16	−0.1 (4)
N1—C8—C9—C18	−70.5 (3)	C11—C12—C17—C16	179.0 (2)

### Hydrogen-bond geometry (Å, °)

**Table:**

*D*—H···*A*	*D*—H	H···*A*	*D*···*A*	*D*—H···*A*
O1—H1···N1	0.81	1.88	2.593 (3)	147
O2—H2···N2	0.83	1.90	2.604 (3)	143
